# Customizable Self-Microemulsifying Rectal Suppositories by Semisolid Extrusion 3D Printing

**DOI:** 10.3390/pharmaceutics16111359

**Published:** 2024-10-24

**Authors:** Hye Jin Park, Dong Wuk Kim

**Affiliations:** BK21 FOUR Community-Based Intelligent Novel Drug Discovery Education Unit, Vessel-Organ Interaction Research Center (VOICE, MRC), Research Institute of Pharmaceutical Sciences, College of Pharmacy, Kyungpook National University, Daegu 41566, Republic of Korea; phj051224@naver.com

**Keywords:** SMEDDS suppository, ibuprofen, semisolid extrusion 3D printing, dissolution profiles, customized suppositories

## Abstract

**Objectives**: This study aims to create an innovative self-microemulsifying drug delivery system (SMEDDS) suppository for ibuprofen (IBU) using semisolid extrusion (SSE) three-dimensional (3D) printing technology. **Methods**: Based on solubility studies and the ability to form a transparent microemulsion upon dilution, a selected oil, surfactant, and co-surfactant were utilized to prepare SMEDDS-3DPS containing IBU. The optimal formulation consisted of 10% Triacetin, 80% Gelucire 48/16, and 10% Tetraethylene glycol. SSE 3D printing was employed to create three different-sized suppositories with varying drug contents. These suppositories were assessed for their physicochemical properties, content uniformity, and dissolution profiles. **Results**: The prepared mixture exhibited suitable physical properties for printing, with nano-sized emulsion droplets providing a large surface area for improved drug absorption in the rectum. Characterization techniques such as differential scanning calorimetry, powder X-ray diffraction, and Fourier transform infrared spectroscopy indicated that IBU was present in the formulation in an amorphous state. Additionally, in vitro dissolution tests demonstrated that SMEDDS-3DPS had a significantly higher initial dissolution rate compared with IBU powder. **Conclusions**: This research suggests that SMEDDS-3DPS, as a rectal IBU dosage form, can enhance the rectal bioavailability of IBU. It demonstrates the versatility of 3D printing as a novel manufacturing method for lipid-based suppositories and highlights the simplicity and adaptability of SSE 3D printing technology in producing customized suppositories tailored to individual patient needs, surpassing traditional methods.

## 1. Introduction

Ibuprofen (IBU), a nonselective cyclooxygenase (COX) inhibitor and a nonsteroidal anti-inflammatory drug (NSAID), is commonly used for the treatment of fever, pain, rheumatoid arthritis, and osteoarthritis [1]. According to the Biopharmaceutical Classification System (BCS) [2], a system that classifies drugs into four classes based on solubility in aqueous solution and intestinal permeability [3], IBU is evaluated as a BCS II drug. IBU is typically administered orally, but because of its low water solubility (0.03–2.5 mg/mL, 30 °C) and dissolution rate, it has poor gastrointestinal absorption [4]. Therefore, prodrug [5], inclusion complex [6], microencapsulation [7], oil-based formulation [8], and solid dispersion [9,10] have been developed to enhance the oral absorption and bioavailability of IBU. NSAIDs are known to cause gastrointestinal bleeding, epigastric pain, heartburn, adverse renal effects, and abdominal discomfort when used orally over an extended period of time [8]. And oral administration is sometimes impossible, especially among patients with nausea, vomiting, or unconsciousness [11]. The development of formulations through the rectal route has therefore been attempted in an effort to improve bioavailability and reduce side effects for patients [12].

The rectal route can be considered a good alternative to the oral route for both local and systemic action. Drugs with the following characteristics have advantages when administered rectally [13,14]:

- Low solubility, stability, and permeability.
- Limited absorption, degradable, or unstable in the upper gastrointestinal tract.
- Cause irritation to the gastric mucosa.
- Show a high first-pass metabolism.

Suppositories are generally made by dissolving a suppository base, mixing a drug, injecting the molten material into a suppository mold, cooling the suppository to solidify, and then taking the suppository out of the mold [13,15]. Molding techniques are used in the production of suppositories, and they involve several steps and relatively long solidification times [16]. Even though the molding process seems simple, some steps and parameters must also be carefully examined to prevent problems such as inhomogeneity and crystallization of the drug on the surface of the dosage form. For instance, it is important to consider the appropriate temperature while pouring into the suppository mold because, if the temperature difference between the melt temperature and the mold temperature is too large, the suppository may crack and affect its homogeneity [15]. Furthermore, since this process uses a mold, only fixed-size suppositories can be produced. However, the diameter, length, and volume of the rectum differ by age. The length of the rectum is 3 cm in newborns, 7 cm in children aged 1 year, 9 cm in children aged 6, and 12 cm in children aged 10; the rectum reaches adult size at around age 10 [17]. In the case of rectal drug administration, younger patients exhibit increased drug absorption because of the reduced thickness of the rectal wall, which is comparable to the reduced thickness of external skin [15,17]. Therefore, important factors to be considered when administering suppositories are the patient’s rectal length, diameter, thickness, and surface area. However, conventional suppositories limit various sizes, as well as dose adjustments.

In the pharmaceutical industry, 3D printing technology offers the opportunity to make significant technological advancements in the design and manufacturing of medicine [18]. In contrast to the development and production methods of existing medicines, this technology has the advantage of being able to produce several types of drugs rapidly with multiple materials, which makes it possible to develop medicines that are suitable for individual patient needs and drug release profiles [18,19]. Several formulations are being developed using different 3D printing technologies, such as oral controlled release formulations [20], microneedles [21], implants [22], oral films [23], and suppository molds with various geometric structures. There is a study on manufacturing suppository shells with various numbers and locations of holes for controlled drug release [24], and there is literature comparing different drug dissolution profiles by designing hollow-type suppository shells with different thicknesses and infill percentages [25]. Despite the fact that many studies have been conducted on the development of suppository shells using 3D printers, few studies have been conducted on the development of lipid-based suppositories themselves using this technology.

Some formulation strategies should be used in order to enhance the solubility of poorly soluble drugs and increase rectal absorption. One of them is the self-microemulsifying drug delivery system (SMEDDS), which is one of the most common methods to increase the solubility of poorly soluble drugs. It is an isotropic mixture of oil, surfactant, co-surfactant, and drug that forms a kinetically stable oil-in-water (O/W) emulsion. Because of the fact that lipophilic drugs are dissolved in small lipid droplets with large interfacial surface areas, drug absorption is increased and mucosal damage is decreased [26]. The development of SMEDDS suppositories using 3D printing was initially investigated as a treatment for ulcerative colitis. This demonstrated that the versatility of lipid excipients could affect the disintegration time and drug release kinetics [16]. The focus of 3DP interest in pharmaceutics, however, has mostly been on polymer-based drug delivery systems. There is currently very little literature utilizing 3DP for the development of lipid-based manufacturing.

This study aims to fabricate personalized lipid-based suppositories using the semisolid extrusion (SSE) 3D printing method, developing three types of IBU-3DPSs by varying their dimensions to accommodate different drug contents and sizes. We hypothesize that 3D printing can effectively create customized suppositories that maintain desirable physicochemical properties and meet specific patient requirements. The physicochemical characteristics of the formulations were evaluated by using differential scanning calorimetry (DSC), powder X-ray diffraction (PXRD), Fourier transform infrared spectroscopy (FTIR), scanning electron microscopy (SEM), and transmission electron microscopy (TEM) techniques. In addition, a disintegration and an in vitro dissolution were evaluated in accordance with the *European Pharmacopoeia* (Ph. Eur.) guidelines for suppository formulations [27]. This research highlights the potential of 3D printing as an innovative pharmaceutical manufacturing technology. Ultimately, our study emphasizes the capability of personalized medicine to improve patient care and medication adherence.

## 2. Material and Methods

### 2.1. Materials

Ibuprofen was purchased from the Tokyo Chemical Industry Co. (Tokyo, Japan). Oleic oil, Triacetin (2,3-diacetyloxypropyl acetate), olive oil, castor oil, Tween 20 (polysorbate 20), Tween 80 (polysorbate 80), Tetraethylene glycol, and Polyethylene glycol (PEG) 300 were purchased from Daejung Chemical Co. (Siheung, Republic of Korea). Labrafil M 2130 CS (lauroyl polyoxyl-6 glycerides), Geloil SC (mixture of refined soybean oil, glyceryl distearate and polyglyceryl-3 dioleate), Gelucire 48/16 (polyoxyl-32 stearate), Gelucire 44/14 (lauroyl Polyoxyl-32 glycerides), Labrasol (caprylocaproyl polyoxyl-8 glycerides), and Transcutol P (diethylene glycol monoethyl ether) were donated by Gattefosse (Saint-Priest Cedex, France). Cremophor RH 40 (PEG-40 hydrogenated castor oil), Cremophor EL (PEG-35 castor oil), Kolliphor P 188 (polyethylene-polypropylene glycol), and Kollisolv GTA (glycerol triacetate) were obtained from BASF (Ludwigshafen, Germany). All the other chemicals were of reagent grade and were utilized without additional purification.

### 2.2. Solubility Test

Investigations were performed on the saturation solubility of IBU in aqueous solutions, oils, and surfactants. Double-distilled water was selected as an aqueous solution. Oleic oil, Labrafil M 2130 CS, Triacetin, Geloil SC, olive oil, and castor oil were used as oils, while Gelucire 48/16, Gelucire 44/14, Cremophor RH 40, Cremophor EL, Tween 20, Tween 80, Labrasol, Tetraethylene glycol, Transcutol P, PEG 300, Kolliphor P 188, and Kollisolv GTA were used as surfactants. An excess amount of IBU was added to each centrifuge tube that contained 1 mL of aqueous solution, oil, and surfactant. Mixtures were shaken for 96 h at 40 °C in a water bath shaker. To collect the supernatant, each sample was centrifuged at 10,000 rpm for 20 min using a Microcentrifuge (Hanil, Daejeon, Republic of Korea) after reaching equilibrium. The supernatant was diluted with acetonitrile for quantification of IBU by using the high-performance liquid chromatography (HPLC) method. The drug content was analyzed using HPLC (Thermo Fisher Scientific, Bremen, Germany) that consisted of a UV detector and a reverse-phased C18 column (5 μm, 4.6 mm id × 150 mm, Seongnam, Republic of Korea). The mobile phase consisted of acetonitrile–acidified water (0.7 mL of H_3_PO_4_ in 1000 mL water) = 50:50 (*v*/*v*); the sample injection amount was 10 μL, and the column temperature was maintained at 30 °C. The eluent was monitored at 223 nm with a flow rate of 1.0 mL/min. Prior to conducting the solubility test, a calibration curve was prepared using HPLC under the conditions described above. The curve was generated over a concentration range of 1.95 to 500 µg/mL (n = 3). The resulting calibration equation was y = 0.7505x + 0.5763, with a coefficient of determination R^2^ > 0.999.

### 2.3. Screening of Oil Based on an Emulsification Study

The oil and co-surfactant were selected in addition to their capacity to solubilize the drug through solubility evaluation by measuring the % transmittance and ease of emulsification of the formed microemulsion [28]. Briefly, 300 mg of the selected oil was added to 300 mg of the selected surfactant in a glass vial. The mixture was slowly heated at 50 °C for 20 min to homogenize the mixture, and 50 mg of the isotropic mixture was diluted in 50 mL with double-distilled water in order to obtain a fine emulsion in the stoppered conical tube. By repeatedly inverting the stoppered conical tube, the turbidity of the resulting emulsion was determined to estimate the ease of emulsion formation. After the emulsion was allowed to stand for 2 h, the % transmittance of the microemulsion was measured at 638.2 nm by using a double-beam UV spectrophotometer (UV-1900, Shimadzu, Kyoto, Japan) using double-distilled water as blank.

### 2.4. Screening of Co-Surfactant Based on an Emulsification Study

The emulsifying ability of various co-surfactants was screened in order to find a suitable co-surfactant for the selected oil and surfactant. In a glass vial, 100 mg of the co-surfactant was mixed with 200 mg of the selected surfactant. Then, 300 mg of the selected oil was added to this mixture, which was homogenized and gently heated at 50 °C for 20 min. Next, 50 mg of the isotropic mixture was added to 50 mL with water to obtain a fine emulsion in the stoppered conical tube. The emulsion was allowed to stand for 2 h, as described in the previous section, and transmittance was measured at 638.2 nm by using a UV spectrophotometer.

### 2.5. Construction of a Pseudoternary Phase Diagram

A study on the existence of self-emulsifying fields was conducted to determine the self-emulsifying system formation area for different ratios of oil, surfactant, and co-surfactant. Based on the solubility test results and emulsification studies, Triacetin, Gelucire 48/16, and Tetraethylene glycol were selected as the oil, surfactant, and co-surfactant, respectively. The mixtures in different proportions (0.2 mL) were poured into 200 mL of distilled water in a glass beaker, heated to 37 °C, and stirred gently using a magnetic stirrer. The tendency to spontaneously emulsify and the progress of the emulsion droplets were observed. The tendency of microemulsion was classified as “good” when droplets smoothly spread in water without phase separation, and it was classified as “bad” when there was insufficient or no emulsion formulation with the immediate coalescence of oil droplets, especially when stirring was terminated [29]. A pseudoternary phase diagram was constructed based on the results. All studies were carried out three times, with similar observations being made between repeats.

### 2.6. Preparation of IBU-Loaded SMEDDS

Using the pseudoternary phase diagram self-emulsifying region, the SMEDDS formulation was determined. The optimal formulation selected for further formulation analysis was based on visual assessment and particle size analysis. The formulation of SMEDDS was manufactured by dissolving IBU into 5 mL of a mixture of Triacetin, Gelucire 48/16, and Tetraethylene glycol, as shown in Table 1. The oil, surfactant, and co-surfactant were vortexed for 40 min at 50 °C (mixture melting point) until a clear solution was reached. Then, 10 *w*/*w*% of IBU was added to the well-mixed oil mixture and stirred at 78 °C (IBU melting point) for 30 min. After making sure that the materials were thoroughly combined, the liquid mixture was poured into a 3D printing syringe-type cavity and allowed to solidify at room temperature for about 2 h.

### 2.7. Fabrication of SMEDDS-IBUS Using Semisolid Extrusion 3D Printing

The formerly prepared mixture, which had solidified at room temperature, was used to fabricate suppositories by using a commercial semi-solid extrusion-based printer, Dr. INVIVO 4D6 (Rokit Healthcare, Seoul, Republic of Korea). The SSE 3D printer functions by rapidly melting a semi-solid material contained in a syringe at the appropriate temperature and then solidifying it on the bed to form the desired object. IBU-3DPS was designed using Tinkercad (Autodesk Inc., San Rafael, CA, USA) and comes in three different sizes (X = 5 mm, Y = 5 mm, and Z = 20 mm for 50 mg suppositories; X = 6 mm, Y = 6 mm, and Z = 28 mm for 100 mg suppositories; and X = 8 mm, Y = 8 mm, and Z = 34 mm for 200 mg suppositories). The designed file was saved as a stereolithography (.stl) file in NewCreatorK version 1.57.70 (Rokit Healthcare, Seoul, Republic of Korea). The following were the other printing conditions, optimized through multiple preliminary experiments: 100% infill percentage, 21 G (0.50 mm) nozzle size, 33 °C nozzle temperature, 0.45 mm layer height, 15% infill overlap, 5 mm/s print speed, 5 mm/s travel speed, 0.50 mm shell thickness, and 4 °C platform temperature. Successfully fabricated suppositories were stored at 4 °C after printing until further testing.

### 2.8. Analysis of Physicochemical Characteristics

#### 2.8.1. Morphological Analysis

SEM images of the shape and surface of IBU were taken using a scanning electron microscope (SU-8220; Hitachi, Tokyo, Japan) operating at an accelerated voltage of 5.0 kV. Before analysis, the sample was attached to a brass specimen holder using double-sided adhesive tape to make the sample electrically conductive, and it was then coated with platinum (6 nm/min) in a vacuum (0.8 Pa) for 4 min at 15 mA using an EmiTech Sputter Coater K575 K (Quorum Technologies Ltd., West Sussex, U.K.).

A transmission electron microscope (Hitachi HT 7700; Hitachi, Tokyo, Japan) was used to observe the droplet morphology of SMEDDS. The sample was diluted with double-distilled water at a ratio of 1:100 and stirred gently. A drop of the diluted sample was dropped on the carbon-coated grid. The excess liquid was removed, and the sample was analyzed after drying for 5 min at room temperature.

#### 2.8.2. Droplet Size and Zeta Potential Analysis

The droplet size and zeta potential of SMEDDS were determined using a Zetasizer Nano S9 (Malvern Instrument; Malvern, U.K.) dynamic light scattering particle size analyzer at room temperature at a scattering angle of 90°. The prepared SMEDDS was diluted with double-distilled water at a ratio of 1:100, and then 1 mL of the sample was taken and analyzed. All experiments were performed three times, and the reading of the z-average diameter was used.

#### 2.8.3. Differential Scanning Calorimetry

A DSC (Q20; TA Instruments, Newcastle, DE, USA) was used to examine the thermal properties of IBU, Triacetin, Gelucire 48/16, Tetraethylene glycol, IBU-3DPS, and PM (with IBU). Approximately 5 mg of the sample was weighed, sealed, and put in DSC aluminum pans by heating up from −20 °C to 100 °C at a rate of 10 °C/min. Measurement data were collected and analyzed using TA 2000 Universal Analysis software, and all experiments were performed in triplicate.

#### 2.8.4. Powder X-Ray Diffraction

A powder X-ray diffractometer (D/MAX-2500; Rigaku; Tokyo, Japan) equipped with Cu K radiation (1.54178 Å, 40 kV, and 40 mA) was utilized to record the physical morphology of the powder samples (IBU, Gelucire 48/16, IBU-3DPS, and PM). At room temperature and with a step size of 0.05°/s, the diffraction profiles were obtained in the 2θ range from 5° to 60°.

#### 2.8.5. Fourier Transform Infrared Spectroscopy

Using a Fourier transform infrared spectroscope (Cary630 FTIR, Agilent Technologies, Santa Clara, CA, USA), the molecular interactions among IBU, Triacetin, Gelucire 48/16, and Tetraethylene glycol were examined. The sample was scanned using 16 scans at a resolution of 2 cm^−1^ from 400 to 4000 cm^−1^.

#### 2.8.6. Calculating the Size and Weight Variation of SMEDDS-IBUS

In order to ensure that the suppository was well manufactured as designed, the diameter and height of the suppository were measured using a vernier caliper, and the average and deviation were also assessed (n = 20). Additionally, 20 suppositories were randomly selected, and the uniformity of their weight was evaluated using an electronic scale. If less than two samples had an excess of more than 5% and did not exceed 10% of the average mass, the suppositories were deemed suitable for the uniformity mass test [23].

#### 2.8.7. Determination of Softening and Melting Points

Each sample of suppository was placed in a conical tube with a thermometer inserted. The entire suppository was submerged below the water level by holding the tube vertically and then immersing it in a water bath to a depth of approximately 8 cm. The temperature of the water bath was gradually increased (1 °C/2 min). The softening point was determined to be the point at which the suppository started to melt, while the melting point was the point at which the suppository completely melted. The result is the average value of a total of three repetitions.

#### 2.8.8. Determination of Disintegration Time

SMEDDS-3DPS was tested based on the procedure described in the *European Pharmacopoeia*. According to the *European Pharmacopoeia*, lipophilic suppositories must disintegrate within 30 min, meaning that all their components are completely dissolved [30]. Six randomly selected suppositories were placed in an apparatus (ERWEKA, Heusenstamm, Germany) that consisted of six cylindrical glass tubes, each containing 160 mL of distilled water in a water bath that was kept at a temperature of 37 °C ± 1 °C. A disk was placed in each glass tube to keep the suppository from floating, and the time for each suppository to completely disintegrate was recorded.

#### 2.8.9. Analysis of the Drug Contents in SMEDDS-3DPS 

Content analysis was carried out to determine if the drug was evenly dissolved in the IBU-3DPS formulations. Each of the three formulations was properly diluted with 100 mL of mobile phase and shaken until completely dissolved in a clear volumetric flask. The solution was filtered using a 0.45 μm nylon syringe filter, and the drug content test was carried out using the HPLC method.

Drug ontents (%) = Ca/Cb × 100

where Ca represents the actual amount of drug present in the IBU-3DPS formulation and Cb represents the theoretical amount of drug present in the IBU-3DPS formulation.

### 2.9. In Vitro Dissolution Study

A USP type I dissolution apparatus (DT 620; ERWEKA, Heusenstamm, Germany) was used to evaluate the in vitro drug release of IBU powder (100 mg) and 3D-printed suppositories (IBU content: 50 mg, 100 mg, 200 mg). The studies were carried out using 900 mL of phosphate buffer (pH 7.2) at a temperature of 38 °C ± 0.5 °C, and the paddle speed was maintained at 50 rpm. At predetermined intervals (0, 5, 10, 15, 30, 45, and 60 min), samples (3 mL) were extracted, and an equal volume of fresh solution was added immediately to compensate for any losses during sampling. The collected samples were filtered through 0.45 μm polytetrafluoroethylene membrane syringe filters and analyzed under the HPLC conditions described previously. After conducting a dissolution test for each formulation, the average and deviation were measured (n = 6).

### 2.10. Data and Statistical Analysis

Data were expressed as means ± standard deviations. The student’s *t*-test was used to compare two different groups of samples. A *p*-value less than 0.05 was considered statistically significant.

## 3. Results and Discussion

### 3.1. Selection of the Carriers

#### 3.1.1. Selection of the Surfactant

**Figure 1:**
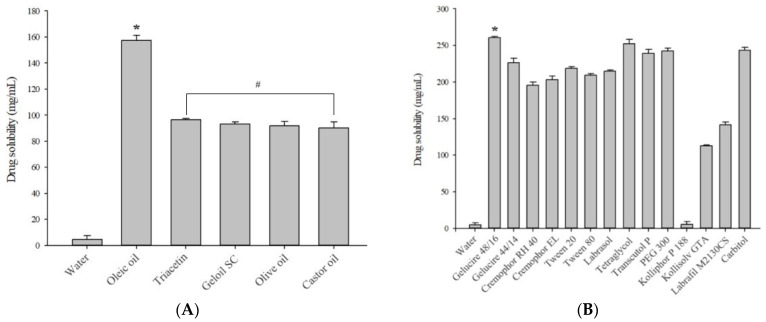
Solubility of IBU in aqueous solution, oils (**A**), and surfactants (**B**) (n = 3). * *p* < 0.05 when compared to all other samples. ^#^ *p* > 0.05 when compared to each other.

SMEDDS, an isotropic liquid mixture of oils, surfactants, co-surfactants, and drugs, is widely used to enhance the bioavailability of drugs by increasing their solubility [31]. This system should form a fine emulsion in the aqueous phase by mild agitation. To achieve optimal drug loading, it is also crucial to identify oils, surfactants, and co-surfactants with a good drug-solubilizing ability [32]. Therefore, solubility tests were carried out in aqueous solutions, oils, and surfactants to select an SMEDDS composition suitable for IBU (Figure 1). Among the various tested surfactants, Gelucire 48/16 solubilized the most drug (262.25 ± 1.86 mg/mL), and the results of the Student’s *t*-test confirmed a significant difference compared with all the other samples (*p* < 0.05). Gelucire 48/16, a saturated medium-chain fatty acid ester with an HLB of 14, has been widely used in the pharmaceutical industry to solubilize poorly soluble drugs and enhance bioavailability [33]. It can stay solid at room temperature (1–23 °C) and transition to a semisolid or liquid state as the temperature increases. Gelucire 48/16 is therefore widely used in studies on suppositories that are solid at room temperature but easily soften when inserted into the rectum [26]. Hence, Gelucire 48/16 was selected as the surfactant serving as the matrix for the formulation of SMEDDS-based suppositories, based on its excellent solubilization capacity and temperature-dependent phase transition properties.

#### 3.1.2. Selection of the Oil

In further studies, in addition to its ability to dissolve IBU, the selection of oil or co-surfactant was governed by its emulsification efficiency. The solubility of IBU in oil is generally taken into consideration when selecting an oil for an SMEDDS formulation. This is due to the fact that the better the solubility of the drug, the higher the drug loading potential [34]. Among the oils that were tested, the solubility of the drug in oleic oil was significantly higher compared with the other oils (*p* < 0.05). However, it was confirmed that oleic oil was not adequately emulsified in Gelucire 48/16, the surfactant selected for the % transmittance test (Table 2). With the exception of oleic oil, all the screened oils showed similar solubility for IBU (*p* > 0.05), but Triacetin in particular was able to solubilize the highest amount of the drug (96.58 mg/mL), which is almost 21 times higher than the reported thermodynamic solubility of IBU in water (4.58 mg/mL). Triacetin had the highest % transmittance performance, indicating that Gelucire 48/16 has good emulsification efficiency for Triacetin. Furthermore, Triacetin was selected as the oil because of its widespread use and favorable rheological behavior, as well as the fact that it is not toxic [35].

#### 3.1.3. Selection of the Co-Surfactant

Co-surfactants provide the surfactant layer sufficient flexibility to increase the area to form the microemulsion [36]. Table 3 presents the results of the % transmittance obtained using various co-surfactants with the previously selected surfactants and oils. The ability of every co-surfactant to emulsify the oil phase was found to be excellent (>90%), with the exception of Kollisolv GTA. Surfactants with higher HLB values usually have better emulsification ability. This is probably due to the hydrophilicity property of the surfactant, which makes it possible for the oil to disperse quickly and easily in the aqueous phase. However, it has been noted that the results of % transmittance do not exactly match the HLB value of the surfactant. In other words, emulsification is not only affected by the structure and chain length of the surfactant but also by other factors. Non-ionic surfactants are generally considered safer than ionic surfactants and have been shown to provide microemulsions with better stability over a wider pH and ionic strength range [37]. Tetraethylene glycol, a non-ionic surfactant with the best drug-solubilizing ability, was therefore selected as a co-surfactant. Tetraethylene glycol is an effective solubilizer, widely used in formulations because of its ability to solubilize various drugs and provide excellent droplet size and emulsion storage stability [38]. Lastly, Triacetin, Gelucire 48/16, and Tetraethylene glycol were selected as the oil, surfactant, and co-surfactant, respectively, based on the results of solubilization and emulsification efficiency. All selected excipients have numerous documented uses and are generally recognized as safe, suggesting that they are unlikely to exhibit toxicity toward the rectal mucosa.

### 3.2. Preparation of IBU-Loaded SMEDDS

**Figure 2:**
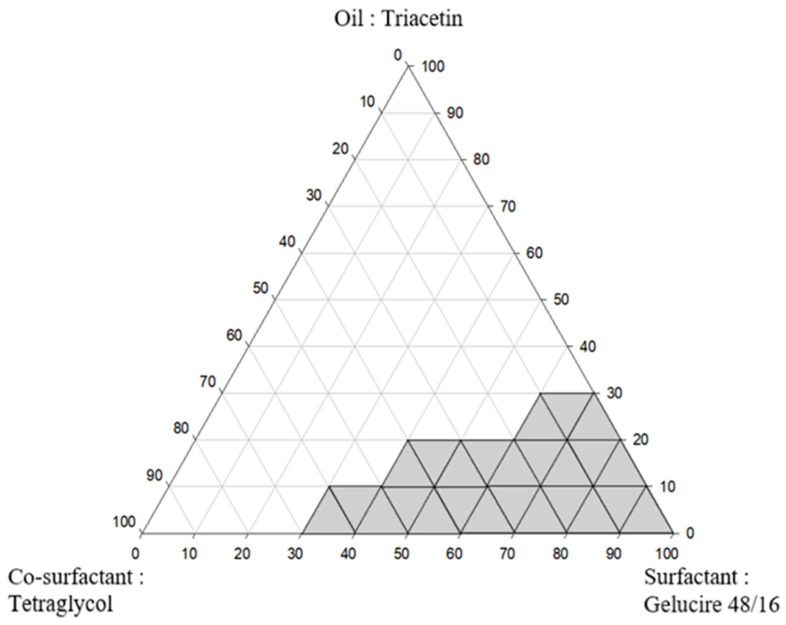
Pseudoternary phase diagram of the oil (Triacetin), surfactant (Gelucire 48/16), and co-surfactant (Tetraethylene glycol).

Firstly, the self-emulsifying properties of the selected oil, surfactant, and co-surfactant were visually assessed [39]. To identify the optimized regions of components in SMEDDS, a pseudoternary diagram was constructed without IBU (Figure 2). The composition ratio of each material was set in various ranges from 0% to 100%, and the mixture was adequately vortexed to prepare formulations of SMEDDS. The prepared formulation was added dropwise to double-distilled water to evaluate the ability to form self-emulsification. The light gray areas indicate stable self-emulsifying regions with a fine milky white color. When the oil was between 5% and 30%, spontaneous emulsion formulation was demonstrated to form an emulsion with a clear or slightly blue or white appearance. A cloudy emulsion was formed as the proportion of oil gradually increased. When the oil ratio was 40% or more, it was found that the formed emulsion had extremely high turbidity and a large droplet size. Clear emulsions did not form in the 5–30% oil ratio range when the ratio of co-surfactant was high, but emulsions did form well when the ratio of surfactant was high. It was also proven that when the co-surfactant ratio was high, the droplet size was large even when a clear emulsion was formed. One of the factors affecting the bioavailability of the drug is the z-average diameter of the manufactured SMEDDS emulsion [29]. In the formulations of SMEDDS, it is well known that the higher the surfactant ratio, the more the interface is stabilized and condensed, and that the higher the co-surfactant ratio, the more the interface expands [40]. Triacetin 10%, Gelucire 48/16 80%, and Tetraethylene glycol 10% were selected as the optimal SMEDDS production ratio considering the self-emulsifying area, droplet size, and safety area during manufacturing. A suppository was created using a 3D printer from the prepared formulation, and several features were assessed.

### 3.3. Analysis of Physicochemical Characteristic

#### 3.3.1. Morphological Analysis

**Figure 3:**
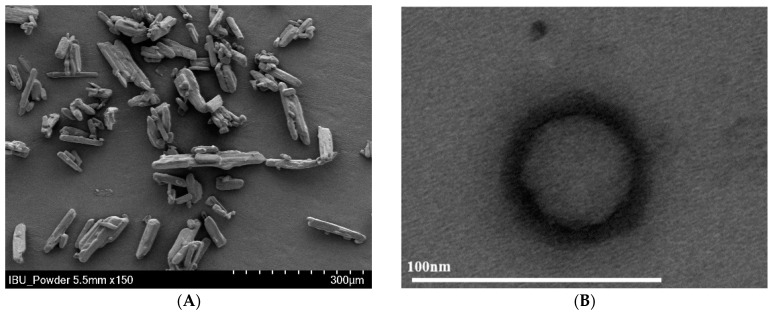
Scanning electron micrograph of IBU (**A**) and transmission electron micrograph of the SMEDDS formulation (**B**).

The IBU particle morphology observed through SEM is shown in Figure 3A. IBU exhibited a thin, long shape with a particle size range of 100 to 150 μm. The morphology of the SMEDDS formulation in water was observed using TEM (Figure 3B). The droplet was spherical in shape and had a well-maintained nano-size. This demonstrates that SMEDDS was stable and well-prepared. Images of dispersed emulsion droplets were not obtained because of the formation of an oily film by Gelucire 48/16 at room temperature. Additionally, it is well known that when a drug is dispersed in oil, the oil covers the surface of the drug as it dissolves in the oil, helping to improve the shape of the drug particle [41]. It can be expected that the long IBU particles will be distributed into spherical droplets after the application of the SMEDDS formulation, improving drug solubility and stability.

#### 3.3.2. Droplet Size and Zeta Potential Analysis

The average droplet size of SMEDDS was 65.91 ± 1.88 nm, indicating that the droplets in the formulation were nano-sized. The prepared SMEDDS formed less than 100 nm microemulsion, which is the standard of SMEDDS. Additionally, a small droplet size ensures improved drug release and better stability of the dispersion system [42]. When compared to TEM, the result of droplet size showed no significant difference. The polydispersity index (PDI) value was measured as 0.10. A low PDI value of 0.2 or less indicates that the particles are in a very homogeneous state [43]. Thus, it can be stated that the SMEDDS formulation contains uniformly distributed nano-sized droplets.

The zeta potential of SMEDDS was −10.15 ± 1.59 mV. The zeta potential is a value for the emulsion’s surface charge and is an index related to the stability of an emulsion. The droplets of the emulsion do not clump together and are well distributed because of the repulsive force between them. The zeta potential of SMEDDS formulations is generally reported to be greater than 10 mV in absolute value to ensure formulation stability [44]. Consequently, it was determined that the prepared SMEDDS had a small z-average particle size and a uniform, physically stable microemulsion.

#### 3.3.3. Differential Scanning Calorimetry

**Figure 4:**
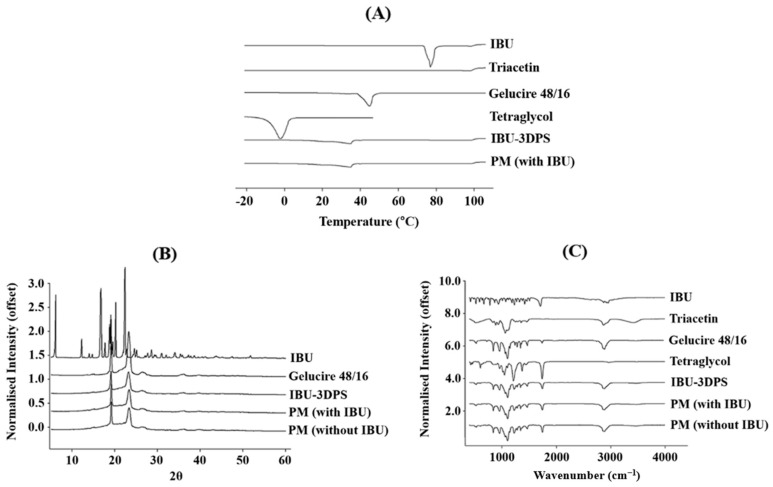
DSC (**A**), PXRD (**B**), and FTIR (**C**) images of samples.

Figure 4A presents the DSC thermograms of IBU, excipients (Triacetin, Gelucire 48/16, Tetraethylene glycol), IBU-3DPS, and PM (with IBU). According to the results of the DSC thermogram, IBU had a sharp single endothermic peak at approximately 74 °C–83 °C, indicating its melting point [45]. Triacetin exhibited a small endothermic peak, while Gelucire 48/16 and Tetraethylene glycol showed a strong endothermic peak at 43 °C–44 °C and −2 °C, respectively. These results were produced by progressive moisture loss [46]. Observing the characteristic peaks of IBU in IBU-3DPS and PM was challenging. It is clear from the DSC thermogram results that IBU is amorphously dispersed within the 3DPS because there are no peaks in those results. Similarly, the thermogram of PM indicates that during the physical mixing process, IBU is distributed within the matrix, leading to a conversion to an amorphous status. In addition, complementary investigations were carried out using PXRD to assess the true physical state of IBU and excipients to further understand the observed changes.

#### 3.3.4. Powder X-Ray Diffraction

IBU powder, excipients (Gelucire 48/16), IBU-3DPS, and PM (with IBU and without IBU) were evaluated by using PXRD. IBU powder characteristic diffraction peaks were observed at 6, 11, 19, 20, and 22° (2θ diffraction angle), as shown in Figure 4B [45]. This result demonstrates that IBU has intrinsic crystallinity on XRD patterns. Gelucire 48/16 exhibited sharp diffraction peaks, especially at 19 and 20° (2θ diffraction angle) [47]. Surprisingly, in the IBU-3DPS and PM (with IBU) results, the sharp IBU peak was not observed, and only the Gelucire 48/16 peaks were maintained. This finding was further supported by evaluating PM (without IBU). The peaks of PM (without IBU) are very similar to those of IBU-3DPS and PM (with IBU), indicating that the IBU-3DPS and PM (with IBU) peaks are excipient peaks, not IBU diffraction peaks. This shows that IBU is distributed in the matrix in an amorphous condition during the mixing process with lipid excipients at 74 °C, which is IBU’s melting temperature when preparing SMEDDS.

#### 3.3.5. Fourier Transform Infrared Spectroscopy

All samples were analyzed, and FTIR spectroscopy was also used to determine compatibility between the IBU-selected lipid excipients and any potential molecular interactions among the formulation components. Figure 4C shows the characteristic FTIR spectra for IBU powder at 668 cm^−1^ (C-H stretching), 779 cm^−1^ (CH2 rocking vibration), 1231 cm^−1^ (C–C stretching), 1721 cm^−1^ (C = O stretching vibrations), and 2920^−1^ (OH stretching) [48]. The majority of IBU powder peaks in the IBU-3DPS and PM (with IBU) results were weakened, shifted, broadened, or disappeared. This indicates that there were some weak interactions, such as hydrogen bonds, between the lipid excipients selected for solubilization and IBU [49]. Similar to previous DSC and PXRD results, the pattern of IBU-3DPS that contained IBU and the pattern of PM (without IBU) are comparable, indicating that IBU crystalline substances do not exist in the final formulation.

#### 3.3.6. Geometry and Physical Characteristics of SMEDDS-IBUS

**Figure 5:**
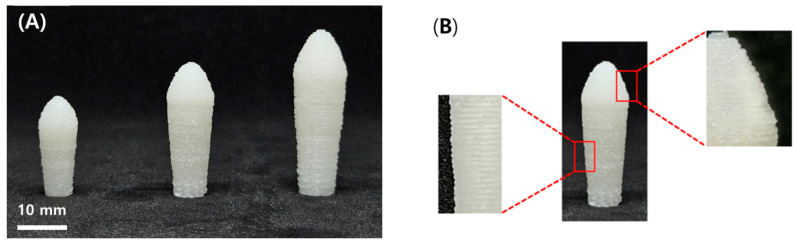
Picture of IBU-3DPS printed in three different sizes (from left to right: 50 mg, 100 mg, and 200 mg) (**A**) and different sections of IBU-3DPS (**B**).

The appearance of a suppository manufactured using 3D printing is depicted in Figure 5A. In this study, we aimed to assess the potential and feasibility of SSE 3D printing by developing patient-specific suppositories containing specific doses of drugs in various sizes. The prepared suppository had a uniform milky white color and was layered without cracks, as shown in Figure 5B. This proved that a suppository can be manufactured using a 3D printer with a selected drug dose in a single-step process without the need for a mold.

Table 4 shows the dimensions of IBU-3DPS, including its diameter, height, weight, softening point, melting point, and disintegration time. Each suppository was designed as follows (diameter × height): IBU-3DPS-50 mg was 5 mm × 20 mm, IBU-3DPS-100 mg was 6 mm × 28 mm, and IBU-3DPS-200 mg was 8 mm × 34 mm. Similar results were found when comparing the diameter and height of the designed suppository and the manufactured suppository. It was also confirmed that the manufactured suppositories were produced reproducibly since the diameter and height deviations were small. Furthermore, it was determined that the prepared suppository was appropriate for the uniform mass test because it was prepared to have sufficient repeatability to ensure that no more than two samples would exceed 5% and 10% of the average mass.

A melting temperature greater than 20 °C is recommended since the suppository must maintain its shape during insertion into the rectum and under storage conditions. To avoid uncomfortable pressure and ensure rapid release of the drug, the suppositories should also dissolve in the rectum as quickly as possible after application. Thus, the narrower the softening and melting point ranges, the better. The prepared suppository was determined to have an appropriate range based on the findings of the obtained softening point and melting point.

The suppository completely disintegrated within the 30-minute time limit set by the *European Pharmacopoeia* for lipophilic suppositories [27].

#### 3.3.7. Analysis of the Drug Contents in SMEDDS-3DPS 

A content test was carried out to make sure that the drug was homogeneously distributed in the SMEDDS-3DPS formulation. The samples used in the experiments were obtained from each printed tablet at the initial, intermediate, and final points. By using the mobile phase, the samples were then diluted to an appropriate concentration. The test was repeated three times in total. IBU-3DPS in dosages of 50 mg, 100 mg, and 200 mg resulted in drug contents of 99.89 ± 0.09, 100.51 ± 0.15, and 98.99 ± 0.31, respectively. This demonstrated that the drug was evenly distributed throughout the SMEDDS formulation and did not precipitate or separate when the SMEDDS formulation containing IBU was put into the syringe-type cavity and solidified.

### 3.4. In Vitro Dissolution Test

**Figure 6:**
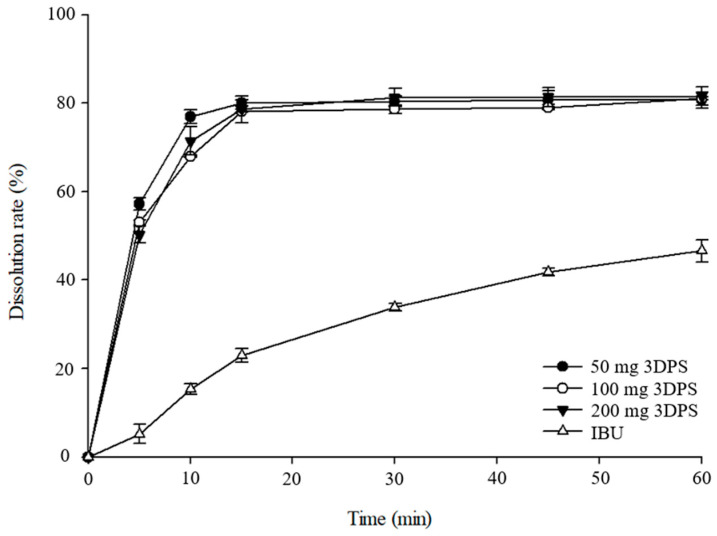
Dissolution profile of IBU powder and 3DPS (50 mg, 100 mg, and 200 mg) in pH 7.2 buffer (n = 6).

The in vitro dissolution tests for IBU powder and SMEDDS-3DPS were monitored for 1 h at pH 7.4 (Figure 6). Given the limited fluid volume in the rectal area, using a small amount of medium can effectively mimic the dissolution environment of the human body. However, since the primary purpose of this dissolution test was to compare different-sized 3DPSs, the test was conducted using 900 mL of medium to account for the laboratory environment. Effective SMEDDS has moderate polarity when it comes into contact with the aqueous phase, which causes the drug to be rapidly released from the oil droplet into the aqueous phase [49]. SMEDDS-3DPS reached the highest dissolution rate within 15 min after the initiation of the dissolution test, as shown in Figure 6. After that, the dissolution curve maintained a saturated state. When compared with the raw material, it is clear that the manufactured SMEDDS-3DPS has a significantly higher dissolution rate. SMEDDS-3DPS has a higher dissolution rate than IBU powder because, as indicated by the DSC, PXRD, and FTIR results, IBU exists in an amorphous state within the SMEDDS matrix. As a result, its solubility in the dissolution medium is significantly increased. Furthermore, the delivery of drugs from the intestinal lumen to the rectal mucosa could be limited by their ability to dissolve and diffuse through mucus [13]. Since the droplet size of SMEDDS is smaller than that of IBU powder, the drug’s surface area is increased, and its solubility is enhanced by the use of the oil, surfactant, and co-surfactant, increasing diffusion into the medium. The prepared formulation exhibited high dissolution behavior right from the start of the experiment, demonstrating that SMEDDS-3DPS may be rapidly dispersed when inserted into the rectum. Subsequently, it is reasonable to expect that the drug has rapid absorption into the rectal mucosa and improved bioavailability.

## 4. Conclusions

By using SSE 3DP, SMEDDS suppositories of IBU were successfully developed. The oil (Triacetin), surfactant (Gelucire 48/16), and co-surfactant (Tetraethylene glycol) were selected through solubility and emulsification tests. Through self-emulsification assessment, a pseudoternary phase diagram was created, and the final formulation was prepared using 10% of Triacetin, 80% of Gelucire 48/16, and 10% of Tetraethylene glycol. The prepared mixture exhibited physical properties suitable for printing and nano-sized emulsion droplets (65.91 ± 1.88 nm), which provided a large surface area for improved drug absorption in the rectum. SMEDDS-3DPS was produced with various drug compositions and sizes, and it exhibited good stacking without any cracks or breaks. In addition, the in vitro dissolution test of the prepared suppository showed that its dissolution profile was better than that of IBU powder; thus, it is expected that when injected into the rectum, the drug will be rapidly absorbed via the rectal mucosa. Hence, this study shows the versatility of 3DP as a new manufacturing method for manufacturing lipid-based suppositories and shows the feasibility of using SSE 3D printing technology to fabricate patient-specific suppositories in a single-step process without the need for molds.

## Figures and Tables

**Table 1:** Selected composition of SMEDDS according to the solubility and self-emulsifying assessment.

	Composition	Amount
Oil	Triacetin	0.5 mL
Surfactant	Gelucire 48/16	4.0 mL
Co-surfactant	Tetraethylene glycol	0.5 mL

**Table 2:** Solubility of IBU in various oils and their emulsification efficiencies.

Oil	Solubility (mg/mL)	% Transmittance
Oleic oil	157.35 ± 6.08	20.2 ± 2.3
Triacetin	96.58 ± 1.09	100.8 ± 0.1
Geloil SC	93.08 ± 2.01	30.6 ± 5.4
Olive oil	91.86 ± 4.36	89.3 ± 1.3
Castor oil	90.09 ± 7.21	88.3 ± 0.9

**Table 3:** Solubility of IBU in various co-surfactants and their emulsification efficiencies.

Co-Surfactant	Solubility (mg/mL)	% Transmittance
Labrasol	214.72 ± 1.55	90.3 ± 0.9
Tetraethylene glycol	252.25 ± 5.86	100.1 ± 0.1
Transcutol P	239.10 ± 5.21	99.7 ± 0.9
PEG 300	242.51 ± 3.59	100.1 ± 0.2
Kolliphor P 188	5.31 ± 4.03	100.2 ± 0.1
Kollisolv GTA	112.87 ± 1.08	14.1 ± 1.5
Labrafil M2130CS	141.61 ± 3.68	100.3 ± 0.1
Carbitol	243.70 ± 3.57	100.2 ± 0.1

**Table 4:** Physical properties of IBU 3D-printed suppositories.

Formulation	Physical Properties of 3DPS					
	Diameter (mm)	Height (mm)	Weight (mg)	Softening Point (°C)	Melting Point (°C)	Disintegration Time (min)
IBU-3DPS—50 mg	4.83 ± 0.70	19.07 ± 0.62	495.63 ± 2.90	31.0 ± 0.00	36.3 ± 0.20	5.34 ± 0.28
IBU-3DPS—100 mg	5.86 ± 0.40	27.43 ± 0.53	997.99 ± 2.12	31.3 ± 0.60	36.8 ± 0.00	5.12 ± 0.70
IBU-3DPS—200 mg	7.60 ± 0.26	33.90 ± 0.16	1496.20 ± 1.61	32.4 ± 0.50	37.1 ± 0.10	4.47 ± 0.52

## Data Availability

The original contributions presented in the study are included in the article, further inquiries can be directed to the corresponding author.
